# Bis[1,3-bis­(1-benzyl-1*H*-benzimidazol-2-yl)-2-oxapropane]nickel(II) dipicrate–dimethyl­formamide–ethanol (1/2/0.25)

**DOI:** 10.1107/S1600536809021163

**Published:** 2009-06-10

**Authors:** Huilu Wu, Ruirui Yun, Ke Li, Kaitong Wang, Xingcai Huang

**Affiliations:** a School of Chemical and Biological Engineering, Lanzhou Jiaotong University, Lanzhou 730070, People’s Republic of China

## Abstract

In the title compound, [Ni(C_30_H_26_N_4_O)_2_](C_6_H_2_N_3_O_7_)_2_·2C_3_H_7_NO·0.25CH_3_CH_2_OH, the Ni^II^ ion is coordinated in a distorted octa­hedral environment by four N atoms and two O atoms from two tridendate 1,3-bis­(1-benzyl-1*H*-benzimidazol-2-yl)-2-oxapropane ligands. The crystal structure is stabilized by weak inter­molecular C—H⋯O hydrogen bonds and weak π–π stacking inter­actions [centroid–centroid distance 3.501 (3) Å]. As well as the cation, two anions and two dimethyl­formamide solvent mol­ecules, the asymmetric unit also contains an ethanol solvent molecule with 0.25 occupancy.

## Related literature

For background to the applications of bis­(2-benzimidazol­yl)alkanes and their derivatives, see: Hendriks *et al.* (1982 ▶); Piquet *et al.* (1995 ▶); Roderick *et al.* (1972 ▶); Wu *et al.* (2005 ▶); van Berkel *et al.* (1995 ▶).
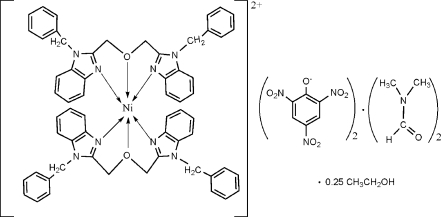


## Experimental

### 

#### Crystal data

[Ni(C_30_H_26_N_4_O)_2_](C_6_H_2_N_3_O_7_)_2_·2C_3_H_7_NO·0.25C_2_H_6_O
*M* *_r_* = 1589.73
Monoclinic, 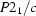

*a* = 16.6265 (4) Å
*b* = 18.1735 (5) Å
*c* = 26.6287 (8) Å
β = 107.513 (1)°
*V* = 7673.2 (4) Å^3^
*Z* = 4
Mo *K*α radiation
μ = 0.34 mm^−1^
*T* = 153 K
0.16 × 0.12 × 0.11 mm

#### Data collection

Rigaku R-AXIS SPIDER diffractometer
Absorption correction: multi-scan (*ABSCOR*; Higashi, 1995 ▶) *T*_min_ = 0.948, *T*_max_ = 0.964
57512 measured reflections
13620 independent reflections
7422 reflections with *I* > 2σ(*I*)
*R*_int_ = 0.100

#### Refinement

*R*[*F* ^2^ > 2σ(*F* ^2^)] = 0.093
*wR*(*F* ^2^) = 0.290
*S* = 1.14
13620 reflections
1031 parameters
33 restraints
H-atom parameters constrained
Δρ_max_ = 1.47 e Å^−3^
Δρ_min_ = −0.70 e Å^−3^

### 

Data collection: *RAPID-AUTO* (Rigaku/MSC, 2004 ▶); cell refinement: *RAPID-AUTO*; data reduction: *RAPID-AUTO*; program(s) used to solve structure: *SHELXS97* (Sheldrick, 2008 ▶); program(s) used to refine structure: *SHELXL97* (Sheldrick, 2008 ▶); molecular graphics: *SHELXTL* (Sheldrick, 2008 ▶); software used to prepare material for publication: *SHELXTL*.

## Supplementary Material

Crystal structure: contains datablocks global, I. DOI: 10.1107/S1600536809021163/lh2826sup1.cif

Structure factors: contains datablocks I. DOI: 10.1107/S1600536809021163/lh2826Isup2.hkl

Additional supplementary materials:  crystallographic information; 3D view; checkCIF report

## Figures and Tables

**Table 1:** Hydrogen-bond geometry (Å, °)

*D*—H⋯*A*	*D*—H	H⋯*A*	*D*⋯*A*	*D*—H⋯*A*
C2—H2*A*⋯O10^i^	0.95	2.47	3.286 (8)	144
C8—H8*A*⋯O5	0.99	2.35	3.337 (9)	173
C8—H8*B*⋯O12^ii^	0.99	2.36	3.185 (9)	140
C9—H9*A*⋯O12^ii^	0.99	2.28	3.125 (9)	142
C9—H9*B*⋯O4	0.99	2.49	3.417 (7)	156
C17—H17*A*⋯O8^ii^	0.99	2.39	3.341 (9)	161
C38—H38*A*⋯O17	0.99	2.17	3.134 (8)	164
C38—H38*B*⋯O10^i^	0.99	2.31	3.072 (7)	133
C39—H39*A*⋯O10^i^	0.99	2.36	3.137 (9)	135
C39—H39*A*⋯O11^i^	0.99	2.35	3.141 (10)	137
C43—H43*A*⋯O16^iii^	0.95	2.46	3.281 (8)	144
C56—H56*A*⋯O11^i^	0.95	2.46	3.346 (11)	155
C65—H65*A*⋯O12	0.95	2.50	3.438 (10)	169
C77—H77*B*⋯O15	0.98	2.50	3.389 (15)	150

Symmetry codes: (i) 
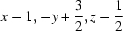
; (ii) 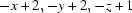; (iii) 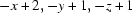.

**Fig. 1.**
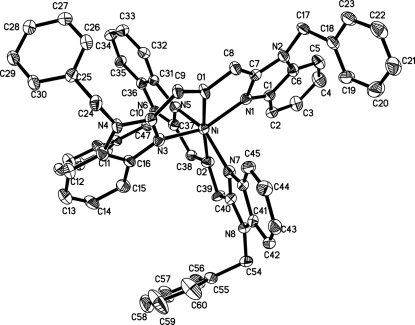
The molecular structure of the cation. Hydrogen atoms have been omitted for clarity and the displacement ellipsoids are shown at the 30% probability level.

**Fig. 2.**
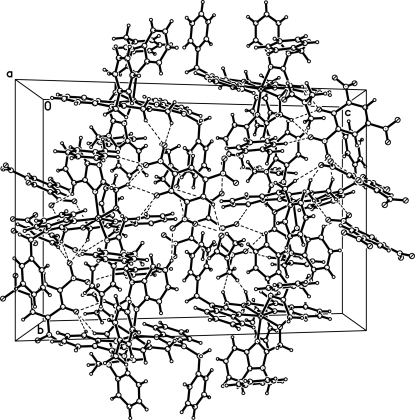
Part of the crystal structure with weak hydrogen bonds shown as dashed lines. For clarity the solvent molecules are not included.

## supplementary crystallographic information

### Comment

There is widespread interest in bis(2-benzimidazolyl)alkanes and their derivatives because of their wide-ranging anti-virus activity (Roderick *et al.*, 1972), their importance in selective ion-exchange resins (van Berkel *et al.*, 1995), and the possibility of forming supramolecular aggregates with d^10^ metal ions in which discrete macrocycles, 1, 2 and 3-D architectures have been generated (Piquet *et al.* 1995). We have been interested in utilizing benzimidazolyl substituted tripodal ligands with nitrogen cores to construct supramolecules, which could provide hydrogen bond donor NH groups and π–π stacking interactions (Hendriks *et al.*, 1982). In our work, efforts are focused on the tridentate ligand, 1,3-bis(1-benzylbenzimidazol-2-yl)-2-oxopropane, which is similar to the histidine imidazole ligand in its coordination aspects (Wu *et al.* 2005). Since the two arms of this type of ligand can each rotate freely about an O(apical)-C bond, multicomponent complexes or coordination polymeric networks may be expected to form from the assembly of this ligand with metal ions of low coordination number. Herein, the crystal structure of the title compound is presented. The molecular structure of the cation is shown in Fig. 1. The Ni^II^ ion is coordinated in a disotorted octahedral environment inolving four N atoms and two O atoms from two tridendate ligands with the axial sites occupied by two oxygen atoms. The crystal structure is stabilized by weak intermolecular C-H···O hydrogen bonds as well as weak π–π stacking interactions with a centroid to centroid distance of 3.501 (3) Å between two benzimidazole ring systems related by the symmetry operator (1-x,-1/2+y,1/2-z).

### Experimental

Reagents and solvents used were of commercially available quality. To a strred solution of 1,3-bis(1-benzylbenzimidazol-2-yl)-2-oxopropane (183.2 mg, 0.4 mmol) in hot MeOH (10 ml) was added Ni(C_6_H_2_N_3_O_7_)_2_ (102.9 mg, 0.2 mmol) in MeOH (5 ml). A green crystalline product formed rapidly. The precipitate was filtered off, washed with methanol and absolute ethanol, and dried *in vacuo*. The dried precipitate was dissolved in DMF to form a green solution that was allowed to evaporate at room temperature. The green crystals suitable for X-ray diffraction studies were obtained after three weeks (Yield, 64%). Elemental analysis - found: C, 59.30; H, 4.51; N, 14.14; calc. for C_78.5_H_71.5_N_16_O_18.25_Ni, C, 59.31; H, 4.53; N, 14.10%.

### Refinement

All H atoms were found in difference electron maps and were subsequently refined in a riding-model approximation with C—H distances ranging from 0.95 to 0.99 Å and *U*_iso_(H) = 1.2 *U*_eq_ or *U*_iso_(H) = 1.5 *U*_eq_ for methyl C atoms. The abundance of solvent which is loosely held in the crystal lattice is probably the reason for the lower than normal precision of this structure.

### Crystal data

**Table:**

[Ni(C_30_H_26_N_4_O)_2_](C_6_H_2_N_3_O_7_)_2_·2C_3_H_7_NO·0.25C_2_H_6_O	*F*(000) = 3314
*M**_r_* = 1589.73	*D*_x_ = 1.376 Mg m^−^^3^
Monoclinic, *P*2_1_/*c*	Mo *K*α radiation, λ = 0.71073 Å
Hall symbol: -P 2ybc	Cell parameters from 7422 reflections
*a* = 16.6265 (4) Å	θ = 3.1–25.3°
*b* = 18.1735 (5) Å	µ = 0.34 mm^−^^1^
*c* = 26.6287 (8) Å	*T* = 153 K
β = 107.513 (1)°	Block, green
*V* = 7673.2 (4) Å^3^	0.16 × 0.12 × 0.11 mm
*Z* = 4	

### Data collection

**Table:**

Rigaku R-AXIS SPIDER diffractometer	13620 independent reflections
Radiation source: fine-focus sealed tube	7422 reflections with *I* > 2σ(*I*)
graphite	*R*_int_ = 0.100
φ and ω scans	θ_max_ = 25.3°, θ_min_ = 3.1°
Absorption correction: multi-scan (*ABSCOR*; Higashi, 1995)	*h* = −19→19
*T*_min_ = 0.948, *T*_max_ = 0.964	*k* = −21→19
57512 measured reflections	*l* = −31→31

### Refinement

**Table:**

Refinement on *F*^2^	Secondary atom site location: difference Fourier map
Least-squares matrix: full	Hydrogen site location: inferred from neighbouring sites
*R*[*F*^2^ > 2σ(*F*^2^)] = 0.093	H-atom parameters constrained
*wR*(*F*^2^) = 0.290	*w* = 1/[σ^2^(*F*_o_^2^) + (0.159*P*)^2^] where *P* = (*F*_o_^2^ + 2*F*_c_^2^)/3
*S* = 1.14	(Δ/σ)_max_ < 0.001
13620 reflections	Δρ_max_ = 1.47 e Å^−^^3^
1031 parameters	Δρ_min_ = −0.70 e Å^−^^3^
33 restraints	Extinction correction: *SHELXL97* (Sheldrick, 2008), Fc^*^=kFc[1+0.001xFc^2^λ^3^/sin(2θ)]^-1/4^
Primary atom site location: structure-invariant direct methods	Extinction coefficient: 0.0065 (7)

### Special details

**Table:**

Geometry. All e.s.d.'s (except the e.s.d. in the dihedral angle between two l.s. planes) are estimated using the full covariance matrix. The cell e.s.d.'s are taken into account individually in the estimation of e.s.d.'s in distances, angles and torsion angles; correlations between e.s.d.'s in cell parameters are only used when they are defined by crystal symmetry. An approximate (isotropic) treatment of cell e.s.d.'s is used for estimating e.s.d.'s involving l.s. planes.
Refinement. Refinement of *F*^2^ against ALL reflections. The weighted *R*-factor *wR* and goodness of fit *S* are based on *F*^2^, conventional *R*-factors *R* are based on *F*, with *F* set to zero for negative *F*^2^. The threshold expression of *F*^2^ > σ(*F*^2^) is used only for calculating *R*-factors(gt) *etc*. and is not relevant to the choice of reflections for refinement. *R*-factors based on *F*^2^ are statistically about twice as large as those based on *F*, and *R*- factors based on ALL data will be even larger.

### Fractional atomic coordinates and isotropic or equivalent isotropic displacement parameters (Å^2^)

**Table:**

	*x*	*y*	*z*	*U*_iso_*/*U*_eq_	Occ. (<1)
Ni1	0.51131 (4)	1.03417 (4)	0.30185 (3)	0.0332 (3)	
O1	0.6244 (2)	1.0747 (2)	0.35372 (15)	0.0425 (10)	
O2	0.4051 (2)	0.9916 (2)	0.24517 (16)	0.0405 (10)	
O3	0.8696 (4)	0.8778 (5)	0.5346 (3)	0.140 (3)	
O4	0.8102 (3)	0.9785 (3)	0.4610 (2)	0.0640 (14)	
O5	0.8510 (3)	0.9599 (4)	0.3932 (3)	0.109 (3)	
O6	1.1420 (3)	0.9104 (3)	0.4209 (2)	0.0726 (15)	
O7	1.2164 (2)	0.8564 (3)	0.49189 (19)	0.0598 (13)	
O8	1.0962 (4)	0.8107 (4)	0.6320 (2)	0.102 (2)	
O9	0.9629 (4)	0.7997 (5)	0.6121 (2)	0.118 (3)	
O10	1.3372 (3)	0.5619 (2)	0.62338 (16)	0.0508 (11)	
O11	1.2770 (5)	0.6866 (4)	0.6542 (3)	0.127 (3)	
O12	1.2726 (4)	0.7690 (4)	0.5996 (3)	0.102 (2)	
O13	1.0995 (3)	0.7107 (3)	0.42298 (18)	0.0639 (14)	
O14	1.1353 (3)	0.6130 (3)	0.38853 (19)	0.0774 (16)	
O15	1.3402 (4)	0.4428 (3)	0.5091 (3)	0.092 (2)	
O17	0.1841 (3)	0.9649 (4)	0.2410 (3)	0.102 (2)	
N1	0.5890 (3)	1.0487 (2)	0.25471 (18)	0.0349 (11)	
N2	0.7160 (3)	1.0756 (3)	0.24688 (18)	0.0366 (11)	
N3	0.4864 (3)	1.0455 (3)	0.37249 (18)	0.0372 (11)	
N4	0.5239 (3)	1.0850 (3)	0.45632 (19)	0.0392 (12)	
N5	0.4393 (3)	1.1266 (2)	0.27307 (18)	0.0362 (11)	
N6	0.3186 (3)	1.1743 (2)	0.22166 (18)	0.0351 (11)	
N7	0.5244 (3)	0.9208 (3)	0.31261 (18)	0.0382 (11)	
N8	0.4661 (3)	0.8095 (2)	0.29733 (19)	0.0372 (11)	
N9	0.8613 (3)	0.9548 (3)	0.4406 (3)	0.0603 (16)	
N10	1.1504 (3)	0.8839 (3)	0.4647 (2)	0.0493 (14)	
N11	1.0251 (4)	0.8202 (4)	0.6018 (2)	0.0683 (17)	
N12	1.2632 (4)	0.7030 (3)	0.6109 (2)	0.0554 (15)	
N13	1.1364 (3)	0.6518 (3)	0.4264 (2)	0.0558 (15)	
N14	1.3027 (4)	0.4623 (3)	0.5403 (3)	0.0678 (19)	
N15	0.0523 (5)	0.9466 (4)	0.1851 (3)	0.097 (3)	
N16	1.2579 (5)	0.3023 (5)	0.4119 (3)	0.093 (3)	
C1	0.5847 (3)	1.0439 (3)	0.2013 (2)	0.0362 (13)	
C2	0.5181 (4)	1.0263 (3)	0.1569 (2)	0.0437 (15)	
H2A	0.4636	1.0152	0.1593	0.052*	
C3	0.5351 (4)	1.0256 (4)	0.1095 (3)	0.0530 (17)	
H3A	0.4906	1.0145	0.0785	0.064*	
C4	0.6155 (4)	1.0407 (4)	0.1048 (3)	0.0606 (19)	
H4A	0.6240	1.0393	0.0711	0.073*	
C5	0.6816 (4)	1.0573 (4)	0.1483 (3)	0.0535 (17)	
H5A	0.7364	1.0670	0.1457	0.064*	
C6	0.6648 (3)	1.0594 (3)	0.1960 (2)	0.0401 (14)	
C7	0.6678 (3)	1.0671 (3)	0.2798 (2)	0.0360 (13)	
C8	0.6983 (3)	1.0797 (3)	0.3371 (2)	0.0401 (14)	
H8A	0.7401	1.0418	0.3547	0.048*	
H8B	0.7247	1.1289	0.3452	0.048*	
C9	0.6348 (3)	1.0839 (3)	0.4085 (2)	0.0397 (14)	
H9A	0.6552	1.1340	0.4203	0.048*	
H9B	0.6751	1.0474	0.4297	0.048*	
C10	0.5476 (3)	1.0716 (3)	0.4128 (2)	0.0390 (14)	
C11	0.4391 (3)	1.0654 (3)	0.4439 (2)	0.0419 (14)	
C12	0.3829 (4)	1.0674 (4)	0.4726 (3)	0.0501 (16)	
H12A	0.3987	1.0845	0.5079	0.060*	
C13	0.3002 (4)	1.0427 (4)	0.4465 (3)	0.0549 (17)	
H13A	0.2592	1.0424	0.4649	0.066*	
C14	0.2779 (4)	1.0189 (4)	0.3948 (3)	0.0578 (19)	
H14A	0.2216	1.0032	0.3785	0.069*	
C15	0.3343 (3)	1.0172 (4)	0.3661 (3)	0.0481 (16)	
H15A	0.3178	1.0005	0.3306	0.058*	
C16	0.4165 (3)	1.0407 (3)	0.3909 (2)	0.0385 (14)	
C17	0.8062 (3)	1.0900 (3)	0.2615 (3)	0.0434 (15)	
H17A	0.8215	1.1241	0.2919	0.052*	
H17B	0.8191	1.1149	0.2318	0.052*	
C18	0.8601 (3)	1.0216 (3)	0.2758 (2)	0.0433 (15)	
C19	0.8303 (4)	0.9522 (3)	0.2611 (3)	0.0555 (19)	
H19A	0.7726	0.9453	0.2419	0.067*	
C20	0.8834 (4)	0.8923 (4)	0.2739 (3)	0.073 (2)	
H20A	0.8614	0.8444	0.2641	0.088*	
C21	0.9670 (4)	0.9005 (4)	0.3004 (3)	0.069 (2)	
H21A	1.0035	0.8591	0.3081	0.083*	
C22	0.9971 (4)	0.9694 (4)	0.3157 (3)	0.064 (2)	
H22A	1.0547	0.9758	0.3351	0.077*	
C23	0.9447 (4)	1.0298 (4)	0.3032 (3)	0.0531 (17)	
H23A	0.9669	1.0775	0.3134	0.064*	
C24	0.5730 (4)	1.1241 (3)	0.5046 (2)	0.0438 (15)	
H24A	0.6340	1.1190	0.5090	0.053*	
H24B	0.5615	1.1020	0.5357	0.053*	
C25	0.5496 (4)	1.2041 (3)	0.5010 (2)	0.0413 (14)	
C26	0.5860 (4)	1.2529 (4)	0.4741 (3)	0.0548 (17)	
H26A	0.6282	1.2362	0.4594	0.066*	
C27	0.5603 (4)	1.3268 (4)	0.4685 (3)	0.0613 (19)	
H27A	0.5846	1.3599	0.4496	0.074*	
C28	0.5001 (4)	1.3513 (4)	0.4903 (3)	0.0496 (16)	
H28A	0.4819	1.4011	0.4856	0.060*	
C29	0.4662 (4)	1.3044 (4)	0.5187 (3)	0.0474 (15)	
H29A	0.4266	1.3223	0.5350	0.057*	
C30	0.4894 (4)	1.2314 (4)	0.5236 (2)	0.0476 (15)	
H30A	0.4643	1.1990	0.5426	0.057*	
C31	0.4383 (3)	1.2021 (3)	0.2832 (2)	0.0339 (13)	
C32	0.4976 (3)	1.2463 (3)	0.3188 (2)	0.0416 (14)	
H32A	0.5484	1.2263	0.3414	0.050*	
C33	0.4793 (4)	1.3192 (3)	0.3195 (3)	0.0471 (15)	
H33A	0.5190	1.3506	0.3429	0.057*	
C34	0.4054 (4)	1.3494 (3)	0.2875 (3)	0.0501 (16)	
H34A	0.3956	1.4007	0.2896	0.060*	
C35	0.3449 (4)	1.3062 (3)	0.2524 (2)	0.0438 (15)	
H35A	0.2934	1.3263	0.2305	0.053*	
C36	0.3642 (3)	1.2319 (3)	0.2512 (2)	0.0384 (13)	
C37	0.3653 (3)	1.1129 (3)	0.2362 (2)	0.0370 (13)	
C38	0.3391 (3)	1.0380 (3)	0.2168 (2)	0.0373 (13)	
H38A	0.2855	1.0245	0.2235	0.045*	
H38B	0.3312	1.0345	0.1785	0.045*	
C39	0.3841 (3)	0.9147 (3)	0.2455 (3)	0.0427 (15)	
H39A	0.3754	0.8924	0.2104	0.051*	
H39B	0.3323	0.9079	0.2560	0.051*	
C40	0.4592 (3)	0.8812 (3)	0.2855 (2)	0.0370 (13)	
C41	0.5432 (3)	0.8010 (3)	0.3368 (2)	0.0372 (13)	
C42	0.5834 (4)	0.7392 (3)	0.3637 (2)	0.0436 (15)	
H42A	0.5591	0.6915	0.3569	0.052*	
C43	0.6609 (4)	0.7510 (3)	0.4012 (2)	0.0464 (15)	
H43A	0.6905	0.7104	0.4208	0.056*	
C44	0.6961 (4)	0.8209 (4)	0.4106 (3)	0.0492 (16)	
H44A	0.7491	0.8269	0.4367	0.059*	
C45	0.6564 (3)	0.8816 (3)	0.3832 (2)	0.0425 (15)	
H45A	0.6814	0.9291	0.3895	0.051*	
C46	0.5778 (3)	0.8709 (3)	0.3456 (2)	0.0372 (13)	
C47	0.2323 (3)	1.1776 (4)	0.1856 (2)	0.0448 (15)	
H47A	0.2219	1.1335	0.1628	0.054*	
H47B	0.2270	1.2214	0.1627	0.054*	
C48	0.1668 (3)	1.1816 (3)	0.2134 (2)	0.0364 (13)	
C49	0.0832 (3)	1.1935 (3)	0.1830 (2)	0.0448 (15)	
H49A	0.0695	1.1976	0.1458	0.054*	
C50	0.0204 (4)	1.1995 (4)	0.2074 (3)	0.0512 (17)	
H50A	−0.0361	1.2089	0.1868	0.061*	
C51	0.0394 (4)	1.1920 (4)	0.2611 (3)	0.0518 (17)	
H51A	−0.0038	1.1963	0.2775	0.062*	
C52	0.1227 (4)	1.1780 (4)	0.2915 (3)	0.0543 (17)	
H52A	0.1362	1.1730	0.3287	0.065*	
C53	0.1853 (3)	1.1716 (4)	0.2672 (2)	0.0473 (16)	
H53A	0.2413	1.1602	0.2877	0.057*	
C54	0.4008 (3)	0.7527 (3)	0.2774 (3)	0.0433 (15)	
H54A	0.4265	0.7033	0.2854	0.052*	
H54B	0.3773	0.7573	0.2387	0.052*	
C55	0.3303 (3)	0.7608 (3)	0.3024 (2)	0.0436 (15)	
C56	0.2494 (4)	0.7785 (4)	0.2713 (3)	0.062 (2)	
H56A	0.2389	0.7836	0.2344	0.074*	
C57	0.1847 (5)	0.7888 (5)	0.2919 (4)	0.079 (2)	
H57A	0.1294	0.7992	0.2698	0.094*	
C58	0.2010 (6)	0.7840 (5)	0.3444 (5)	0.096 (3)	
H58A	0.1567	0.7930	0.3593	0.115*	
C59	0.2789 (6)	0.7664 (6)	0.3768 (4)	0.103 (3)	
H59A	0.2888	0.7634	0.4138	0.124*	
C60	0.3432 (5)	0.7531 (6)	0.3552 (3)	0.085 (3)	
H60A	0.3971	0.7385	0.3773	0.102*	
C61	0.9367 (4)	0.8862 (4)	0.5227 (3)	0.0619 (19)	
C62	0.9366 (3)	0.9175 (4)	0.4735 (3)	0.0503 (17)	
C63	1.0040 (4)	0.9165 (3)	0.4544 (3)	0.0488 (16)	
H63A	0.9999	0.9375	0.4210	0.059*	
C64	1.0792 (3)	0.8844 (3)	0.4846 (2)	0.0429 (15)	
C65	1.0861 (4)	0.8549 (3)	0.5345 (3)	0.0498 (16)	
H65A	1.1380	0.8356	0.5562	0.060*	
C66	1.0154 (4)	0.8550 (4)	0.5510 (3)	0.0504 (16)	
C67	1.2866 (3)	0.5803 (3)	0.5798 (2)	0.0400 (14)	
C68	1.2462 (3)	0.6511 (3)	0.5686 (2)	0.0379 (13)	
C69	1.1993 (3)	0.6749 (3)	0.5199 (2)	0.0411 (14)	
H69A	1.1761	0.7231	0.5150	0.049*	
C70	1.1867 (3)	0.6272 (3)	0.4783 (2)	0.0443 (15)	
C71	1.2208 (4)	0.5562 (4)	0.4845 (3)	0.0499 (16)	
H71A	1.2119	0.5238	0.4553	0.060*	
C72	1.2668 (4)	0.5351 (3)	0.5333 (3)	0.0458 (15)	
C73	0.0782 (11)	0.8842 (10)	0.1631 (7)	0.207 (7)	
H73A	0.1389	0.8767	0.1793	0.311*	
H73B	0.0663	0.8912	0.1251	0.311*	
H73C	0.0476	0.8409	0.1696	0.311*	
C74	−0.0340 (6)	0.9693 (8)	0.1677 (5)	0.147 (6)	
H74A	−0.0400	1.0164	0.1841	0.221*	
H74B	−0.0690	0.9321	0.1776	0.221*	
H74C	−0.0521	0.9751	0.1293	0.221*	
C75	0.1106 (5)	0.9821 (5)	0.2220 (4)	0.081 (3)	
H75A	0.0931	1.0258	0.2352	0.097*	
C76	1.2961 (10)	0.2397 (9)	0.4030 (6)	0.194 (6)	
H76A	1.3401	0.2521	0.3869	0.291*	
H76B	1.2542	0.2075	0.3793	0.291*	
H76C	1.3215	0.2143	0.4365	0.291*	
C77	1.2637 (7)	0.3659 (8)	0.3881 (5)	0.146 (6)	
H77A	1.3035	0.3605	0.3677	0.219*	
H77B	1.2838	0.4043	0.4148	0.219*	
H77C	1.2081	0.3795	0.3646	0.219*	
O16	1.3004 (5)	0.4249 (3)	0.5760 (3)	0.115 (3)	
O18	1.1731 (9)	0.3887 (8)	0.4370 (6)	0.234 (6)	
C78	1.1980 (10)	0.3238 (8)	0.4439 (7)	0.184 (7)	
H78A	1.1816	0.2906	0.4667	0.220*	
C80	0.003 (2)	0.9603 (17)	0.0144 (13)	0.084 (10)*	0.25
H80A	−0.0017	1.0092	−0.0017	0.126*	0.25
H80B	0.0623	0.9459	0.0274	0.126*	0.25
H80C	−0.0211	0.9615	0.0439	0.126*	0.25
C79	−0.046 (2)	0.9045 (15)	−0.0266 (13)	0.094 (11)*	0.25
H79A	−0.1022	0.9258	−0.0427	0.112*	0.25
H79B	−0.0174	0.9040	−0.0545	0.112*	0.25
O19	−0.0592 (19)	0.8280 (16)	−0.0159 (13)	0.137 (11)*	0.25
H19	−0.0162	0.8033	−0.0152	0.206*	0.25

### Atomic displacement parameters (Å^2^)

**Table:**

	*U*^11^	*U*^22^	*U*^33^	*U*^12^	*U*^13^	*U*^23^
Ni1	0.0305 (4)	0.0322 (4)	0.0385 (4)	−0.0002 (3)	0.0124 (3)	−0.0024 (3)
O1	0.0281 (18)	0.066 (3)	0.038 (2)	0.0012 (18)	0.0160 (17)	−0.0047 (19)
O2	0.0346 (19)	0.032 (2)	0.053 (3)	0.0020 (16)	0.0114 (18)	0.0064 (18)
O3	0.102 (5)	0.180 (8)	0.165 (7)	0.053 (5)	0.083 (5)	0.081 (6)
O4	0.041 (2)	0.072 (4)	0.079 (4)	0.012 (2)	0.017 (2)	−0.012 (3)
O5	0.073 (4)	0.187 (7)	0.081 (4)	0.063 (4)	0.042 (3)	0.072 (5)
O6	0.053 (3)	0.099 (4)	0.074 (4)	0.013 (3)	0.032 (3)	0.026 (3)
O7	0.037 (2)	0.074 (3)	0.068 (3)	0.013 (2)	0.016 (2)	0.006 (2)
O8	0.090 (4)	0.118 (5)	0.078 (4)	−0.002 (4)	−0.007 (3)	0.031 (4)
O9	0.090 (4)	0.205 (8)	0.072 (4)	0.002 (5)	0.042 (4)	0.045 (5)
O10	0.058 (3)	0.046 (3)	0.045 (3)	0.009 (2)	0.011 (2)	0.009 (2)
O11	0.212 (8)	0.094 (5)	0.067 (4)	0.071 (5)	0.029 (5)	−0.006 (4)
O12	0.130 (5)	0.079 (5)	0.119 (5)	−0.029 (4)	0.071 (4)	−0.040 (4)
O13	0.065 (3)	0.062 (3)	0.057 (3)	0.018 (2)	0.007 (2)	0.005 (2)
O14	0.086 (4)	0.084 (4)	0.049 (3)	0.020 (3)	0.000 (3)	−0.012 (3)
O15	0.089 (4)	0.079 (4)	0.098 (5)	0.035 (3)	0.012 (4)	−0.025 (3)
O17	0.058 (3)	0.105 (5)	0.149 (6)	0.005 (3)	0.039 (4)	0.018 (4)
N1	0.033 (2)	0.033 (3)	0.036 (3)	−0.0005 (18)	0.008 (2)	−0.003 (2)
N2	0.031 (2)	0.041 (3)	0.041 (3)	0.0010 (19)	0.015 (2)	0.002 (2)
N3	0.035 (2)	0.039 (3)	0.037 (3)	0.0016 (19)	0.009 (2)	−0.007 (2)
N4	0.035 (2)	0.047 (3)	0.040 (3)	0.004 (2)	0.019 (2)	−0.006 (2)
N5	0.038 (2)	0.033 (3)	0.038 (3)	−0.0039 (19)	0.012 (2)	−0.005 (2)
N6	0.033 (2)	0.031 (3)	0.042 (3)	0.0044 (19)	0.011 (2)	0.002 (2)
N7	0.034 (2)	0.040 (3)	0.040 (3)	0.005 (2)	0.011 (2)	0.003 (2)
N8	0.042 (3)	0.029 (3)	0.044 (3)	−0.0049 (19)	0.018 (2)	−0.002 (2)
N9	0.045 (3)	0.062 (4)	0.081 (5)	0.008 (3)	0.029 (3)	0.012 (3)
N10	0.034 (3)	0.056 (4)	0.058 (4)	0.006 (2)	0.015 (3)	0.004 (3)
N11	0.068 (4)	0.090 (5)	0.046 (4)	−0.002 (3)	0.017 (3)	0.007 (3)
N12	0.081 (4)	0.036 (3)	0.045 (3)	0.018 (3)	0.012 (3)	0.005 (3)
N13	0.057 (3)	0.062 (4)	0.043 (3)	0.009 (3)	0.007 (3)	−0.006 (3)
N14	0.077 (4)	0.043 (4)	0.070 (4)	0.019 (3)	0.003 (4)	−0.009 (3)
N15	0.087 (5)	0.074 (5)	0.112 (6)	0.016 (4)	0.004 (5)	−0.005 (5)
N16	0.093 (5)	0.086 (6)	0.075 (5)	−0.003 (4)	−0.011 (4)	−0.019 (4)
C1	0.040 (3)	0.031 (3)	0.036 (3)	0.003 (2)	0.010 (3)	0.002 (2)
C2	0.045 (3)	0.034 (3)	0.054 (4)	−0.003 (2)	0.017 (3)	−0.003 (3)
C3	0.047 (4)	0.071 (5)	0.040 (4)	0.009 (3)	0.013 (3)	0.003 (3)
C4	0.059 (4)	0.085 (6)	0.041 (4)	0.000 (4)	0.019 (3)	0.007 (3)
C5	0.051 (4)	0.066 (5)	0.052 (4)	−0.001 (3)	0.027 (3)	0.009 (3)
C6	0.042 (3)	0.040 (3)	0.040 (3)	0.009 (3)	0.016 (3)	0.008 (3)
C7	0.039 (3)	0.030 (3)	0.041 (3)	0.000 (2)	0.014 (3)	−0.001 (2)
C8	0.034 (3)	0.050 (4)	0.039 (3)	−0.001 (2)	0.014 (3)	−0.005 (3)
C9	0.029 (3)	0.050 (4)	0.040 (3)	0.003 (2)	0.010 (2)	−0.012 (3)
C10	0.036 (3)	0.040 (4)	0.042 (3)	0.004 (2)	0.014 (3)	−0.001 (3)
C11	0.038 (3)	0.043 (4)	0.046 (4)	0.005 (3)	0.015 (3)	0.003 (3)
C12	0.051 (4)	0.061 (4)	0.047 (4)	0.002 (3)	0.027 (3)	−0.002 (3)
C13	0.045 (3)	0.071 (5)	0.056 (4)	0.002 (3)	0.027 (3)	0.005 (3)
C14	0.042 (3)	0.072 (5)	0.065 (5)	−0.014 (3)	0.024 (3)	0.002 (4)
C15	0.039 (3)	0.052 (4)	0.052 (4)	−0.002 (3)	0.012 (3)	−0.001 (3)
C16	0.033 (3)	0.037 (3)	0.049 (4)	−0.001 (2)	0.018 (3)	0.002 (3)
C17	0.030 (3)	0.045 (4)	0.059 (4)	−0.005 (2)	0.019 (3)	0.003 (3)
C18	0.033 (3)	0.049 (4)	0.053 (4)	0.003 (2)	0.020 (3)	0.008 (3)
C19	0.044 (3)	0.038 (4)	0.082 (5)	−0.003 (3)	0.015 (3)	0.004 (3)
C20	0.061 (4)	0.043 (4)	0.111 (7)	0.010 (3)	0.020 (4)	0.010 (4)
C21	0.042 (4)	0.066 (5)	0.100 (6)	0.016 (3)	0.022 (4)	0.015 (4)
C22	0.037 (3)	0.075 (6)	0.078 (5)	0.008 (3)	0.015 (3)	0.024 (4)
C23	0.042 (3)	0.056 (4)	0.066 (5)	−0.008 (3)	0.023 (3)	0.005 (3)
C24	0.043 (3)	0.054 (4)	0.034 (3)	0.004 (3)	0.012 (3)	−0.008 (3)
C25	0.046 (3)	0.039 (3)	0.041 (3)	−0.001 (3)	0.015 (3)	−0.008 (3)
C26	0.057 (4)	0.055 (5)	0.064 (5)	−0.002 (3)	0.035 (3)	−0.012 (3)
C27	0.073 (4)	0.045 (4)	0.077 (5)	−0.016 (3)	0.039 (4)	−0.011 (4)
C28	0.055 (4)	0.047 (4)	0.052 (4)	0.002 (3)	0.023 (3)	−0.006 (3)
C29	0.047 (3)	0.048 (4)	0.054 (4)	0.006 (3)	0.027 (3)	−0.001 (3)
C30	0.048 (3)	0.054 (4)	0.050 (4)	0.002 (3)	0.028 (3)	0.000 (3)
C31	0.032 (3)	0.027 (3)	0.046 (3)	0.003 (2)	0.017 (2)	0.001 (2)
C32	0.039 (3)	0.044 (4)	0.045 (3)	−0.008 (3)	0.018 (3)	−0.001 (3)
C33	0.052 (3)	0.039 (4)	0.053 (4)	−0.007 (3)	0.020 (3)	−0.004 (3)
C34	0.062 (4)	0.028 (3)	0.063 (4)	−0.003 (3)	0.023 (3)	0.000 (3)
C35	0.053 (3)	0.030 (3)	0.051 (4)	0.007 (3)	0.020 (3)	0.004 (3)
C36	0.044 (3)	0.033 (3)	0.043 (3)	0.004 (2)	0.019 (3)	0.004 (3)
C37	0.038 (3)	0.041 (3)	0.032 (3)	−0.002 (2)	0.011 (2)	0.000 (2)
C38	0.032 (3)	0.032 (3)	0.048 (3)	0.004 (2)	0.012 (2)	0.003 (3)
C39	0.042 (3)	0.024 (3)	0.058 (4)	−0.004 (2)	0.010 (3)	−0.007 (3)
C40	0.040 (3)	0.032 (3)	0.042 (3)	−0.001 (2)	0.017 (3)	−0.004 (2)
C41	0.040 (3)	0.031 (3)	0.046 (3)	0.004 (2)	0.021 (3)	−0.003 (2)
C42	0.055 (3)	0.029 (3)	0.057 (4)	0.000 (3)	0.032 (3)	0.004 (3)
C43	0.046 (3)	0.046 (4)	0.052 (4)	0.010 (3)	0.021 (3)	0.011 (3)
C44	0.040 (3)	0.053 (4)	0.051 (4)	−0.001 (3)	0.010 (3)	0.004 (3)
C45	0.035 (3)	0.034 (3)	0.057 (4)	−0.002 (2)	0.011 (3)	0.002 (3)
C46	0.041 (3)	0.028 (3)	0.049 (4)	−0.002 (2)	0.022 (3)	−0.001 (2)
C47	0.032 (3)	0.051 (4)	0.050 (4)	0.004 (3)	0.011 (3)	0.002 (3)
C48	0.031 (3)	0.034 (3)	0.047 (4)	−0.002 (2)	0.016 (3)	0.000 (3)
C49	0.036 (3)	0.052 (4)	0.043 (3)	0.001 (3)	0.007 (3)	−0.004 (3)
C50	0.038 (3)	0.064 (5)	0.049 (4)	0.000 (3)	0.008 (3)	−0.002 (3)
C51	0.037 (3)	0.058 (4)	0.065 (5)	−0.001 (3)	0.022 (3)	−0.003 (3)
C52	0.049 (4)	0.072 (5)	0.044 (4)	0.001 (3)	0.017 (3)	0.003 (3)
C53	0.032 (3)	0.060 (4)	0.050 (4)	0.004 (3)	0.013 (3)	0.011 (3)
C54	0.041 (3)	0.034 (3)	0.056 (4)	−0.009 (2)	0.016 (3)	−0.005 (3)
C55	0.038 (3)	0.041 (4)	0.055 (4)	−0.010 (3)	0.019 (3)	−0.004 (3)
C56	0.049 (4)	0.070 (5)	0.069 (5)	−0.003 (3)	0.020 (4)	0.016 (4)
C57	0.056 (4)	0.087 (7)	0.105 (7)	0.004 (4)	0.042 (5)	0.010 (5)
C58	0.072 (6)	0.103 (8)	0.135 (9)	−0.031 (5)	0.065 (6)	−0.048 (7)
C59	0.092 (6)	0.155 (8)	0.073 (5)	−0.040 (5)	0.041 (5)	−0.026 (5)
C60	0.051 (4)	0.162 (9)	0.035 (4)	−0.023 (5)	0.001 (3)	−0.002 (5)
C61	0.051 (4)	0.082 (6)	0.065 (5)	0.018 (3)	0.037 (4)	0.015 (4)
C62	0.036 (3)	0.051 (4)	0.068 (5)	0.009 (3)	0.022 (3)	0.012 (3)
C63	0.044 (3)	0.046 (4)	0.058 (4)	0.009 (3)	0.018 (3)	0.009 (3)
C64	0.035 (3)	0.046 (4)	0.051 (4)	0.005 (3)	0.017 (3)	0.003 (3)
C65	0.045 (3)	0.047 (4)	0.058 (4)	0.009 (3)	0.017 (3)	0.000 (3)
C66	0.046 (3)	0.059 (4)	0.052 (4)	0.007 (3)	0.023 (3)	0.008 (3)
C67	0.037 (3)	0.037 (3)	0.046 (4)	0.005 (2)	0.013 (3)	0.011 (3)
C68	0.043 (3)	0.033 (3)	0.037 (3)	0.000 (2)	0.010 (3)	−0.002 (2)
C69	0.046 (3)	0.039 (3)	0.034 (3)	0.008 (3)	0.006 (3)	0.005 (3)
C70	0.040 (3)	0.051 (4)	0.035 (3)	0.003 (3)	0.001 (3)	0.004 (3)
C71	0.051 (4)	0.048 (4)	0.047 (4)	0.004 (3)	0.010 (3)	−0.009 (3)
C72	0.050 (3)	0.031 (3)	0.054 (4)	0.004 (3)	0.013 (3)	−0.007 (3)
C73	0.221 (10)	0.175 (10)	0.192 (10)	0.039 (8)	0.010 (8)	−0.020 (8)
C74	0.079 (7)	0.186 (14)	0.148 (12)	0.016 (7)	−0.009 (7)	0.053 (9)
C75	0.066 (5)	0.073 (6)	0.117 (8)	−0.003 (4)	0.047 (5)	0.006 (5)
C76	0.191 (9)	0.159 (9)	0.179 (9)	0.037 (8)	−0.025 (7)	−0.020 (8)
C77	0.127 (9)	0.147 (12)	0.123 (10)	−0.058 (8)	−0.024 (8)	0.053 (9)
O16	0.191 (7)	0.041 (4)	0.102 (5)	0.029 (4)	0.028 (5)	0.016 (3)
O18	0.244 (9)	0.175 (8)	0.243 (9)	0.043 (7)	0.012 (7)	−0.047 (7)
C78	0.166 (9)	0.200 (10)	0.181 (10)	−0.067 (8)	0.046 (8)	0.003 (8)

### Geometric parameters (Å, °)

**Table:**

Ni1—N3	2.055 (5)	C26—C27	1.403 (9)
Ni1—N5	2.070 (5)	C26—H26A	0.9500
Ni1—N1	2.072 (5)	C27—C28	1.374 (9)
Ni1—N7	2.082 (5)	C27—H27A	0.9500
Ni1—O2	2.096 (4)	C28—C29	1.366 (9)
Ni1—O1	2.103 (4)	C28—H28A	0.9500
O1—C9	1.425 (7)	C29—C30	1.378 (9)
O1—C8	1.429 (7)	C29—H29A	0.9500
O2—C38	1.410 (6)	C30—H30A	0.9500
O2—C39	1.440 (6)	C31—C36	1.381 (7)
O3—C61	1.257 (9)	C31—C32	1.396 (7)
O4—N9	1.215 (7)	C32—C33	1.361 (8)
O5—N9	1.225 (8)	C32—H32A	0.9500
O6—N10	1.232 (7)	C33—C34	1.381 (8)
O7—N10	1.224 (6)	C33—H33A	0.9500
O8—N11	1.225 (7)	C34—C35	1.392 (8)
O9—N11	1.207 (8)	C34—H34A	0.9500
O10—C67	1.257 (6)	C35—C36	1.392 (8)
O11—N12	1.146 (8)	C35—H35A	0.9500
O12—N12	1.259 (8)	C37—C38	1.475 (8)
O13—N13	1.224 (7)	C38—H38A	0.9900
O14—N13	1.226 (7)	C38—H38B	0.9900
O15—N14	1.231 (9)	C39—C40	1.504 (8)
O17—C75	1.214 (9)	C39—H39A	0.9900
N1—C7	1.322 (6)	C39—H39B	0.9900
N1—C1	1.405 (7)	C41—C46	1.384 (8)
N2—C7	1.362 (7)	C41—C42	1.392 (8)
N2—C6	1.397 (7)	C42—C43	1.389 (8)
N2—C17	1.455 (6)	C42—H42A	0.9500
N3—C10	1.325 (7)	C43—C44	1.390 (9)
N3—C16	1.393 (7)	C43—H43A	0.9500
N4—C10	1.354 (8)	C44—C45	1.379 (8)
N4—C11	1.394 (7)	C44—H44A	0.9500
N4—C24	1.481 (7)	C45—C46	1.400 (7)
N5—C37	1.347 (6)	C45—H45A	0.9500
N5—C31	1.400 (7)	C47—C48	1.491 (8)
N6—C37	1.348 (7)	C47—H47A	0.9900
N6—C36	1.389 (7)	C47—H47B	0.9900
N6—C47	1.468 (6)	C48—C53	1.385 (8)
N7—C40	1.320 (7)	C48—C49	1.398 (7)
N7—C46	1.384 (7)	C49—C50	1.390 (9)
N8—C40	1.339 (7)	C49—H49A	0.9500
N8—C41	1.399 (7)	C50—C51	1.377 (9)
N8—C54	1.475 (7)	C50—H50A	0.9500
N9—C62	1.461 (8)	C51—C52	1.400 (8)
N10—C64	1.434 (8)	C51—H51A	0.9500
N11—C66	1.457 (9)	C52—C53	1.386 (9)
N12—C68	1.429 (8)	C52—H52A	0.9500
N13—C70	1.454 (7)	C53—H53A	0.9500
N14—O16	1.178 (9)	C54—C55	1.520 (8)
N14—C72	1.442 (8)	C54—H54A	0.9900
N15—C75	1.321 (11)	C54—H54B	0.9900
N15—C73	1.403 (17)	C55—C60	1.364 (9)
N15—C74	1.430 (11)	C55—C56	1.389 (8)
N16—C77	1.334 (12)	C56—C57	1.358 (11)
N16—C76	1.357 (15)	C56—H56A	0.9500
N16—C78	1.545 (19)	C57—C58	1.344 (13)
C1—C2	1.392 (8)	C57—H57A	0.9500
C1—C6	1.409 (8)	C58—C59	1.360 (13)
C2—C3	1.375 (9)	C58—H58A	0.9500
C2—H2A	0.9500	C59—C60	1.380 (12)
C3—C4	1.406 (9)	C59—H59A	0.9500
C3—H3A	0.9500	C60—H60A	0.9500
C4—C5	1.369 (9)	C61—C66	1.417 (9)
C4—H4A	0.9500	C61—C62	1.427 (9)
C5—C6	1.382 (9)	C62—C63	1.364 (9)
C5—H5A	0.9500	C63—C64	1.394 (8)
C7—C8	1.476 (8)	C63—H63A	0.9500
C8—H8A	0.9900	C64—C65	1.406 (9)
C8—H8B	0.9900	C65—C66	1.373 (9)
C9—C10	1.505 (8)	C65—H65A	0.9500
C9—H9A	0.9900	C67—C72	1.439 (8)
C9—H9B	0.9900	C67—C68	1.440 (8)
C11—C12	1.373 (9)	C68—C69	1.368 (7)
C11—C16	1.421 (8)	C69—C70	1.372 (8)
C12—C13	1.415 (9)	C69—H69A	0.9500
C12—H12A	0.9500	C70—C71	1.399 (8)
C13—C14	1.383 (10)	C71—C72	1.351 (9)
C13—H13A	0.9500	C71—H71A	0.9500
C14—C15	1.378 (9)	C73—H73A	0.9800
C14—H14A	0.9500	C73—H73B	0.9800
C15—C16	1.393 (8)	C73—H73C	0.9800
C15—H15A	0.9500	C74—H74A	0.9800
C17—C18	1.513 (8)	C74—H74B	0.9800
C17—H17A	0.9900	C74—H74C	0.9800
C17—H17B	0.9900	C75—H75A	0.9500
C18—C19	1.368 (8)	C76—H76A	0.9800
C18—C23	1.384 (8)	C76—H76B	0.9800
C19—C20	1.379 (9)	C76—H76C	0.9800
C19—H19A	0.9500	C77—H77A	0.9800
C20—C21	1.364 (9)	C77—H77B	0.9800
C20—H20A	0.9500	C77—H77C	0.9800
C21—C22	1.364 (10)	O18—C78	1.245 (3)
C21—H21A	0.9500	C78—H78A	0.9500
C22—C23	1.378 (9)	C80—C79	1.530 (3)
C22—H22A	0.9500	C80—H80A	0.9800
C23—H23A	0.9500	C80—H80B	0.9800
C24—C25	1.500 (8)	C80—H80C	0.9800
C24—H24A	0.9900	C79—O19	1.450 (3)
C24—H24B	0.9900	C79—H79A	0.9900
C25—C26	1.388 (9)	C79—H79B	0.9900
C25—C30	1.403 (8)	O19—H19	0.8400
			
N3—Ni1—N5	90.26 (18)	C31—C32—H32A	121.5
N3—Ni1—N1	151.23 (17)	C32—C33—C34	122.6 (6)
N5—Ni1—N1	93.95 (18)	C32—C33—H33A	118.7
N3—Ni1—N7	90.82 (19)	C34—C33—H33A	118.7
N5—Ni1—N7	151.40 (16)	C33—C34—C35	121.3 (6)
N1—Ni1—N7	98.69 (18)	C33—C34—H34A	119.3
N3—Ni1—O2	109.74 (17)	C35—C34—H34A	119.3
N5—Ni1—O2	76.42 (15)	C34—C35—C36	115.9 (5)
N1—Ni1—O2	98.90 (17)	C34—C35—H35A	122.0
N7—Ni1—O2	76.34 (15)	C36—C35—H35A	122.0
N3—Ni1—O1	75.55 (16)	C31—C36—N6	106.7 (5)
N5—Ni1—O1	105.16 (16)	C31—C36—C35	122.4 (5)
N1—Ni1—O1	75.88 (16)	N6—C36—C35	130.8 (5)
N7—Ni1—O1	102.77 (16)	N5—C37—N6	112.2 (5)
O2—Ni1—O1	174.58 (16)	N5—C37—C38	122.3 (5)
C9—O1—C8	117.2 (4)	N6—C37—C38	125.4 (4)
C9—O1—Ni1	121.5 (3)	O2—C38—C37	105.3 (4)
C8—O1—Ni1	120.1 (3)	O2—C38—H38A	110.7
C38—O2—C39	115.6 (4)	C37—C38—H38A	110.7
C38—O2—Ni1	121.1 (3)	O2—C38—H38B	110.7
C39—O2—Ni1	120.5 (3)	C37—C38—H38B	110.7
C7—N1—C1	105.5 (5)	H38A—C38—H38B	108.8
C7—N1—Ni1	115.5 (4)	O2—C39—C40	104.2 (4)
C1—N1—Ni1	138.9 (3)	O2—C39—H39A	110.9
C7—N2—C6	107.1 (4)	C40—C39—H39A	110.9
C7—N2—C17	127.3 (5)	O2—C39—H39B	110.9
C6—N2—C17	125.2 (5)	C40—C39—H39B	110.9
C10—N3—C16	104.8 (5)	H39A—C39—H39B	108.9
C10—N3—Ni1	117.4 (4)	N7—C40—N8	113.8 (5)
C16—N3—Ni1	137.3 (4)	N7—C40—C39	122.5 (5)
C10—N4—C11	106.6 (5)	N8—C40—C39	123.7 (5)
C10—N4—C24	127.1 (5)	C46—C41—C42	122.9 (5)
C11—N4—C24	125.6 (5)	C46—C41—N8	105.5 (5)
C37—N5—C31	105.0 (4)	C42—C41—N8	131.6 (5)
C37—N5—Ni1	114.9 (4)	C43—C42—C41	116.3 (5)
C31—N5—Ni1	139.7 (3)	C43—C42—H42A	121.8
C37—N6—C36	106.9 (4)	C41—C42—H42A	121.8
C37—N6—C47	126.0 (5)	C42—C43—C44	121.4 (6)
C36—N6—C47	126.8 (4)	C42—C43—H43A	119.3
C40—N7—C46	104.7 (5)	C44—C43—H43A	119.3
C40—N7—Ni1	115.4 (3)	C45—C44—C43	121.9 (5)
C46—N7—Ni1	139.2 (4)	C45—C44—H44A	119.1
C40—N8—C41	106.1 (4)	C43—C44—H44A	119.1
C40—N8—C54	126.4 (4)	C44—C45—C46	117.6 (5)
C41—N8—C54	127.0 (5)	C44—C45—H45A	121.2
O4—N9—O5	122.2 (6)	C46—C45—H45A	121.2
O4—N9—C62	119.0 (7)	N7—C46—C41	109.8 (5)
O5—N9—C62	118.8 (6)	N7—C46—C45	130.2 (5)
O7—N10—O6	123.0 (6)	C41—C46—C45	120.0 (5)
O7—N10—C64	118.6 (6)	N6—C47—C48	113.2 (5)
O6—N10—C64	118.4 (5)	N6—C47—H47A	108.9
O9—N11—O8	122.1 (7)	C48—C47—H47A	108.9
O9—N11—C66	118.8 (6)	N6—C47—H47B	108.9
O8—N11—C66	119.0 (7)	C48—C47—H47B	108.9
O11—N12—O12	119.1 (7)	H47A—C47—H47B	107.8
O11—N12—C68	123.6 (6)	C53—C48—C49	119.4 (5)
O12—N12—C68	116.7 (6)	C53—C48—C47	122.7 (5)
O13—N13—O14	123.9 (6)	C49—C48—C47	117.9 (5)
O13—N13—C70	118.2 (5)	C50—C49—C48	119.9 (6)
O14—N13—C70	117.9 (6)	C50—C49—H49A	120.1
O16—N14—O15	121.7 (7)	C48—C49—H49A	120.1
O16—N14—C72	121.4 (8)	C51—C50—C49	120.5 (6)
O15—N14—C72	116.8 (7)	C51—C50—H50A	119.8
C75—N15—C73	117.1 (9)	C49—C50—H50A	119.8
C75—N15—C74	122.8 (9)	C50—C51—C52	119.8 (6)
C73—N15—C74	120.1 (11)	C50—C51—H51A	120.1
C77—N16—C76	122.7 (14)	C52—C51—H51A	120.1
C77—N16—C78	101.1 (11)	C53—C52—C51	119.7 (6)
C76—N16—C78	136.1 (12)	C53—C52—H52A	120.2
C2—C1—N1	131.3 (5)	C51—C52—H52A	120.2
C2—C1—C6	119.7 (6)	C48—C53—C52	120.6 (5)
N1—C1—C6	109.1 (5)	C48—C53—H53A	119.7
C3—C2—C1	116.8 (6)	C52—C53—H53A	119.7
C3—C2—H2A	121.6	N8—C54—C55	110.9 (5)
C1—C2—H2A	121.6	N8—C54—H54A	109.5
C2—C3—C4	122.9 (6)	C55—C54—H54A	109.5
C2—C3—H3A	118.5	N8—C54—H54B	109.5
C4—C3—H3A	118.5	C55—C54—H54B	109.5
C5—C4—C3	120.7 (7)	H54A—C54—H54B	108.1
C5—C4—H4A	119.6	C60—C55—C56	117.5 (7)
C3—C4—H4A	119.6	C60—C55—C54	122.6 (6)
C4—C5—C6	116.7 (6)	C56—C55—C54	119.9 (6)
C4—C5—H5A	121.6	C57—C56—C55	122.3 (8)
C6—C5—H5A	121.6	C57—C56—H56A	118.9
C5—C6—N2	131.6 (6)	C55—C56—H56A	118.9
C5—C6—C1	123.1 (5)	C58—C57—C56	118.3 (8)
N2—C6—C1	105.3 (5)	C58—C57—H57A	120.8
N1—C7—N2	112.9 (5)	C56—C57—H57A	120.8
N1—C7—C8	122.8 (5)	C57—C58—C59	122.1 (9)
N2—C7—C8	124.3 (5)	C57—C58—H58A	119.0
O1—C8—C7	104.5 (4)	C59—C58—H58A	119.0
O1—C8—H8A	110.9	C58—C59—C60	119.1 (9)
C7—C8—H8A	110.9	C58—C59—H59A	120.5
O1—C8—H8B	110.9	C60—C59—H59A	120.5
C7—C8—H8B	110.9	C55—C60—C59	120.7 (7)
H8A—C8—H8B	108.9	C55—C60—H60A	119.7
O1—C9—C10	103.7 (4)	C59—C60—H60A	119.7
O1—C9—H9A	111.0	O3—C61—C66	124.4 (7)
C10—C9—H9A	111.0	O3—C61—C62	121.6 (7)
O1—C9—H9B	111.0	C66—C61—C62	113.2 (6)
C10—C9—H9B	111.0	C63—C62—C61	124.2 (6)
H9A—C9—H9B	109.0	C63—C62—N9	115.8 (6)
N3—C10—N4	114.0 (5)	C61—C62—N9	120.0 (6)
N3—C10—C9	120.9 (5)	C62—C63—C64	119.1 (6)
N4—C10—C9	125.1 (5)	C62—C63—H63A	120.5
C12—C11—N4	132.1 (6)	C64—C63—H63A	120.5
C12—C11—C16	122.7 (5)	C63—C64—C65	120.6 (6)
N4—C11—C16	105.2 (5)	C63—C64—N10	119.2 (6)
C11—C12—C13	116.3 (6)	C65—C64—N10	120.2 (5)
C11—C12—H12A	121.9	C66—C65—C64	117.9 (6)
C13—C12—H12A	121.9	C66—C65—H65A	121.0
C14—C13—C12	121.1 (6)	C64—C65—H65A	121.0
C14—C13—H13A	119.4	C65—C66—C61	124.9 (6)
C12—C13—H13A	119.4	C65—C66—N11	115.4 (6)
C15—C14—C13	122.5 (6)	C61—C66—N11	119.6 (6)
C15—C14—H14A	118.8	O10—C67—C72	124.0 (5)
C13—C14—H14A	118.8	O10—C67—C68	124.7 (6)
C14—C15—C16	117.6 (6)	C72—C67—C68	111.1 (5)
C14—C15—H15A	121.2	C69—C68—N12	117.4 (5)
C16—C15—H15A	121.2	C69—C68—C67	125.2 (5)
N3—C16—C15	130.9 (6)	N12—C68—C67	117.2 (5)
N3—C16—C11	109.3 (5)	C68—C69—C70	118.1 (5)
C15—C16—C11	119.8 (6)	C68—C69—H69A	121.0
N2—C17—C18	113.8 (5)	C70—C69—H69A	121.0
N2—C17—H17A	108.8	C69—C70—C71	122.0 (5)
C18—C17—H17A	108.8	C69—C70—N13	118.5 (5)
N2—C17—H17B	108.8	C71—C70—N13	119.5 (6)
C18—C17—H17B	108.8	C72—C71—C70	117.8 (6)
H17A—C17—H17B	107.7	C72—C71—H71A	121.1
C19—C18—C23	118.2 (6)	C70—C71—H71A	121.1
C19—C18—C17	123.3 (5)	C71—C72—C67	125.7 (6)
C23—C18—C17	118.4 (5)	C71—C72—N14	118.5 (6)
C18—C19—C20	120.5 (6)	C67—C72—N14	115.8 (5)
C18—C19—H19A	119.8	N15—C73—H73A	109.5
C20—C19—H19A	119.8	N15—C73—H73B	109.5
C21—C20—C19	121.3 (7)	H73A—C73—H73B	109.5
C21—C20—H20A	119.4	N15—C73—H73C	109.5
C19—C20—H20A	119.4	H73A—C73—H73C	109.5
C20—C21—C22	118.6 (7)	H73B—C73—H73C	109.5
C20—C21—H21A	120.7	N15—C74—H74A	109.5
C22—C21—H21A	120.7	N15—C74—H74B	109.5
C21—C22—C23	120.7 (6)	H74A—C74—H74B	109.5
C21—C22—H22A	119.6	N15—C74—H74C	109.5
C23—C22—H22A	119.6	H74A—C74—H74C	109.5
C22—C23—C18	120.6 (6)	H74B—C74—H74C	109.5
C22—C23—H23A	119.7	O17—C75—N15	127.2 (9)
C18—C23—H23A	119.7	O17—C75—H75A	116.4
N4—C24—C25	110.3 (4)	N15—C75—H75A	116.4
N4—C24—H24A	109.6	N16—C76—H76A	109.5
C25—C24—H24A	109.6	N16—C76—H76B	109.5
N4—C24—H24B	109.6	H76A—C76—H76B	109.5
C25—C24—H24B	109.6	N16—C76—H76C	109.5
H24A—C24—H24B	108.1	H76A—C76—H76C	109.5
C26—C25—C30	118.3 (6)	H76B—C76—H76C	109.5
C26—C25—C24	120.4 (6)	N16—C77—H77A	109.5
C30—C25—C24	121.3 (6)	N16—C77—H77B	109.5
C25—C26—C27	120.0 (6)	H77A—C77—H77B	109.5
C25—C26—H26A	120.0	N16—C77—H77C	109.5
C27—C26—H26A	120.0	H77A—C77—H77C	109.5
C28—C27—C26	120.2 (7)	H77B—C77—H77C	109.5
C28—C27—H27A	119.9	O18—C78—N16	113.5 (16)
C26—C27—H27A	119.9	O18—C78—H78A	123.2
C29—C28—C27	120.3 (6)	N16—C78—H78A	123.2
C29—C28—H28A	119.9	C79—C80—H80A	109.5
C27—C28—H28A	119.9	C79—C80—H80B	109.5
C28—C29—C30	120.2 (6)	H80A—C80—H80B	109.5
C28—C29—H29A	119.9	C79—C80—H80C	109.5
C30—C29—H29A	119.9	H80A—C80—H80C	109.5
C29—C30—C25	121.0 (6)	H80B—C80—H80C	109.5
C29—C30—H30A	119.5	O19—C79—C80	125 (3)
C25—C30—H30A	119.5	O19—C79—H79A	106.1
C36—C31—C32	120.6 (5)	C80—C79—H79A	106.1
C36—C31—N5	109.0 (5)	O19—C79—H79B	106.1
C32—C31—N5	130.3 (5)	C80—C79—H79B	106.1
C33—C32—C31	117.0 (5)	H79A—C79—H79B	106.3
C33—C32—H32A	121.5	C79—O19—H19	109.5

### Hydrogen-bond geometry (Å, °)

**Table:**

*D*—H···*A*	*D*—H	H···*A*	*D*···*A*	*D*—H···*A*
C2—H2A···O10^i^	0.95	2.47	3.286 (8)	144
C8—H8A···O5	0.99	2.35	3.337 (9)	173
C8—H8B···O12^ii^	0.99	2.36	3.185 (9)	140
C9—H9A···O12^ii^	0.99	2.28	3.125 (9)	142
C9—H9B···O4	0.99	2.49	3.417 (7)	156
C17—H17A···O8^ii^	0.99	2.39	3.341 (9)	161
C38—H38A···O17	0.99	2.17	3.134 (8)	164
C38—H38B···O10^i^	0.99	2.31	3.072 (7)	133
C39—H39A···O10^i^	0.99	2.36	3.137 (9)	135
C39—H39A···O11^i^	0.99	2.35	3.141 (10)	137
C43—H43A···O16^iii^	0.95	2.46	3.281 (8)	144
C56—H56A···O11^i^	0.95	2.46	3.346 (11)	155
C65—H65A···O12	0.95	2.50	3.438 (10)	169
C77—H77B···O15	0.98	2.50	3.389 (15)	150

Symmetry codes: (i) *x*−1, −*y*+3/2, *z*−1/2; (ii) −*x*+2, −*y*+2, −*z*+1; (iii) −*x*+2, −*y*+1, −*z*+1.
